# Duodenal Neuroendocrine Tumor Presenting as Iron Deficiency Anemia in an Older Adult

**DOI:** 10.7759/cureus.108649

**Published:** 2026-05-11

**Authors:** Fred Ahmadi, Ghaid Touza, Rana Afram, Saif Affas, Firdous Siddiqui

**Affiliations:** 1 Internal Medicine, Ascension Providence Hospital, Southfield, USA; 2 Gastroenterology, Ascension Providence Hospital, Southfield, USA

**Keywords:** duodenal biopsy, duodenal neuroendocrine tumor, iron deficiency anemia (ida), small bowel malignancy, upper endoscopy

## Abstract

Duodenal neuroendocrine tumors are rare neoplasms that are most often identified incidentally during endoscopic evaluation. They typically follow an indolent course. In older adults, iron deficiency anemia is a common finding that can present with symptoms such as fatigue, dyspnea, and weakness. This warrants thorough evaluation to exclude gastrointestinal sources, including less common malignancies. We report a 70-year-old man with a past medical history of prostate cancer treated with prostatectomy and hypertension who presented with fatigue and decreased exercise tolerance and was found to have iron deficiency anemia. Upper endoscopy revealed segmental mucosal abnormalities in the duodenum, including congestion, erythema, friability, granularity, nodularity, punctate white spots, and altered mucosal texture. Biopsies demonstrated a well-differentiated, grade 1 neuroendocrine tumor with adjacent benign villous mucosa and features of acute and chronic duodenitis with superficial erosion.

Immunohistochemistry showed synaptophysin positivity and chromogranin negativity, with a low mitotic index, suggestive of a neuroendocrine tumor. This case highlights an unusual presentation of a rare malignancy in a duodenal neuroendocrine tumor identified during evaluation of iron deficiency anemia. It emphasizes the importance of comprehensive endoscopic assessment and targeted biopsy in patients with unexplained anemia, including consideration of uncommon gastrointestinal neoplasms.

## Introduction

Duodenal neuroendocrine tumors are rare gastrointestinal neoplasms, accounting for a small proportion of all neuroendocrine tumors [1,2]. They most commonly arise in the gastrointestinal tract, particularly in the small intestine, rectum, appendix, and pancreas, while duodenal origins account for only a small subset. The overall incidence of neuroendocrine tumors has increased substantially over recent decades, with reported incidence rates rising from approximately one per 100,000 individuals in the 1970s to greater than six per 100,000 in more recent population-based studies [2]. They are typically slow-growing and are most often identified incidentally during endoscopic evaluation [2,3]. Despite their indolent nature, these tumors may present with nonspecific symptoms or be detected during evaluation for gastrointestinal blood loss, although presentation with iron deficiency anemia is uncommon [3-5].

Iron deficiency anemia in older adults warrants thorough investigation, as it is frequently associated with chronic gastrointestinal bleeding [6]. While common etiologies must be prioritized, less frequent causes, including uncommon malignancies, should also be considered, particularly in patients with complex medical histories such as prior cancer and radiation exposure. Endoscopic evaluation of the duodenum may reveal a range of mucosal findings, many of which are nonspecific. As a result, careful inspection and targeted biopsy are essential for accurate diagnosis. Histopathologic confirmation, including immunohistochemical staining and tumor grading, remains central to diagnosis and guides subsequent management [7,8].

We present a case of a well-differentiated, grade 1 duodenal neuroendocrine tumor identified during evaluation of iron deficiency anemia in a 70-year-old man with a history of prostate cancer. This case highlights the importance of maintaining a broad differential diagnosis in patients with unexplained anemia and reinforces the role of endoscopic evaluation in identifying rare gastrointestinal neoplasms.

## Case presentation

A 70-year-old man with a history of essential hypertension and prostate adenocarcinoma, previously treated with radical prostatectomy followed by adjuvant pelvic radiation, presented with a six-month history of progressive fatigue, exertional dyspnea, and decreased exercise tolerance. He denied melena, hematochezia, abdominal pain, or other overt signs of gastrointestinal bleeding. He reported mild anorexia without significant weight loss. There was no history of peptic ulcer disease, chronic nonsteroidal anti-inflammatory drug use, or known gastrointestinal disorders, including celiac disease.

On examination, he was hemodynamically stable. Mild conjunctival pallor was noted, with otherwise unremarkable cardiovascular, abdominal, and neurologic examinations. Laboratory evaluation demonstrated microcytic anemia with a hemoglobin of 12.4 g/dL and a mean corpuscular volume of 72.9 fL. Iron studies were consistent with iron deficiency, including a serum iron of 41 mcg/dL and transferrin saturation of 11%. Liver function tests, thyroid studies, and hemolysis markers were within normal limits. Prostate-specific antigen was 0.43 ng/mL. Laboratory findings are summarized in Table 1.

**Table 1:** Laboratory findings at initial presentation

Test	Result	Reference range	Interpretation
Hemoglobin	12.4 g/dL	13.5–17.5 g/dL	Low
Mean corpuscular volume (MCV)	72.9 fL	80–100 fL	Microcytic
Serum iron	41 mcg/dL	60–170 mcg/dL	Low
Transferrin saturation	11%	20–50%	Low
Prostate-specific antigen (PSA)	0.43 ng/mL	<4.0 ng/mL	Within normal limits
Liver function tests	Within normal limits	—	Normal
Thyroid function tests	Within normal limits	—	Normal
Hemolysis markers	Within normal limits	—	No evidence of hemolysis

**Figure 1:**
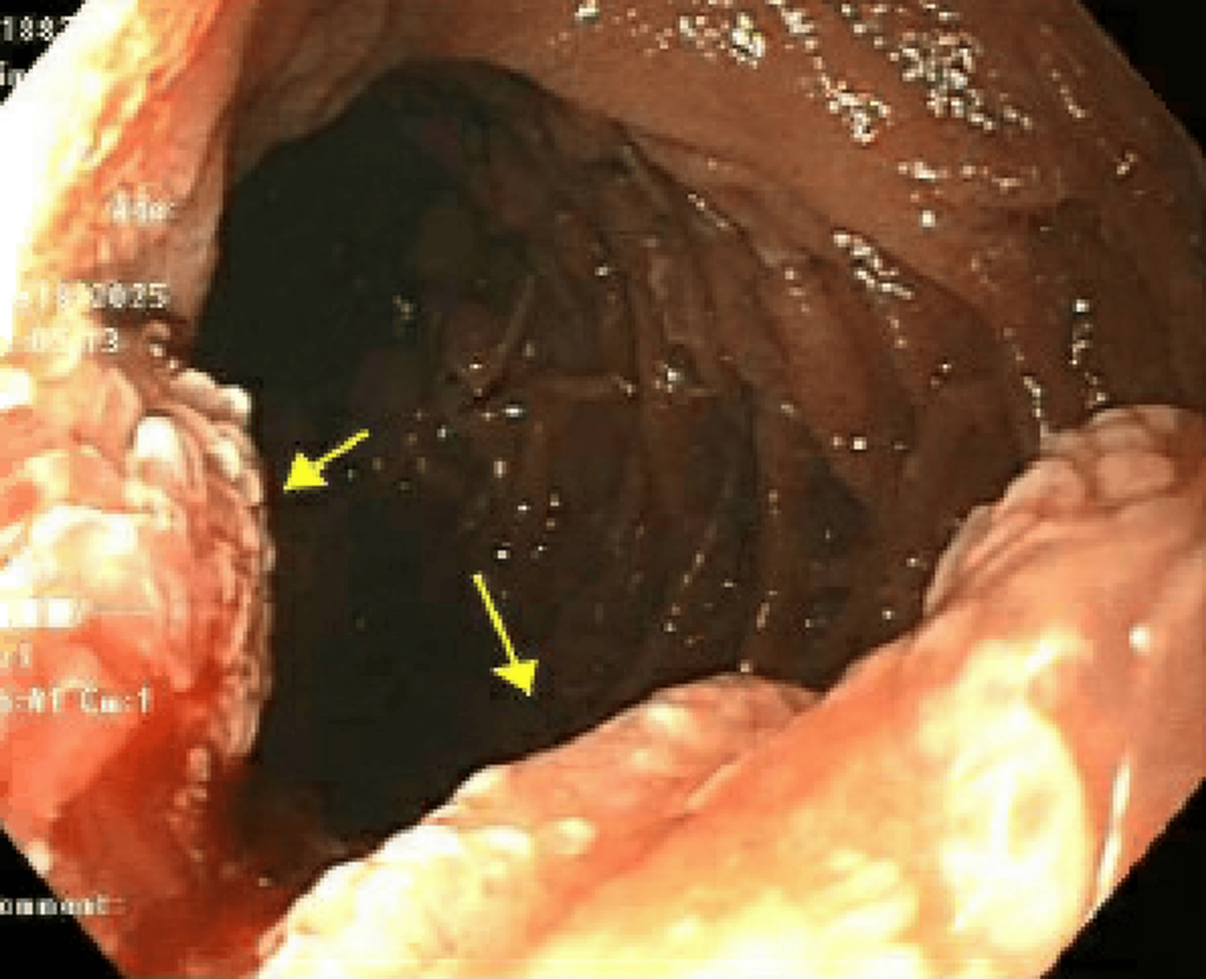
Endoscopic view of the second portion of the duodenum

Given his age and risk factors, evaluation for a gastrointestinal source of blood loss was pursued. A prior flexible sigmoidoscopy performed one year earlier had been unremarkable, demonstrating only non-bleeding internal hemorrhoids. He subsequently underwent esophagogastroduodenoscopy (EGD), which revealed segmental mucosal abnormalities in the second portion of the duodenum characterized by congestion, erythema, friability, granularity, nodularity, punctate white spots, and altered mucosal texture (Figure 1). No active bleeding or discrete mass lesion was identified.

**Figure 2:**
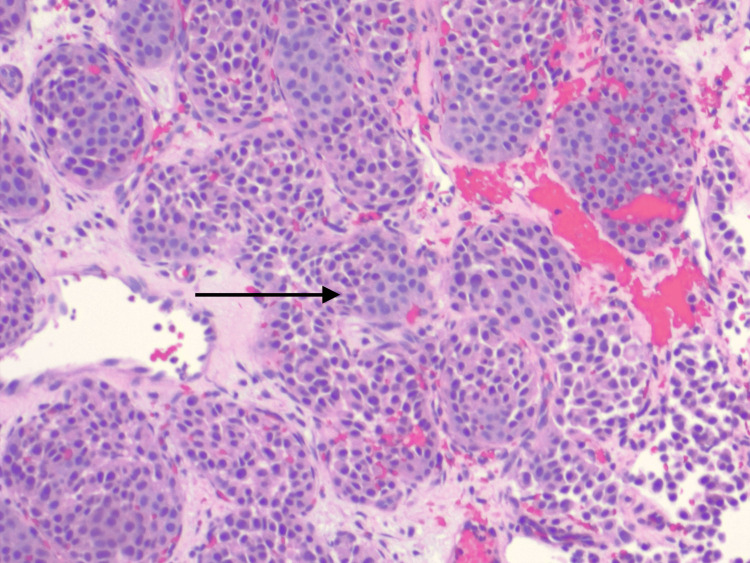
Biopsy demonstrating neuroendocrine tumor cells (100x magnification)

**Figure 3:**
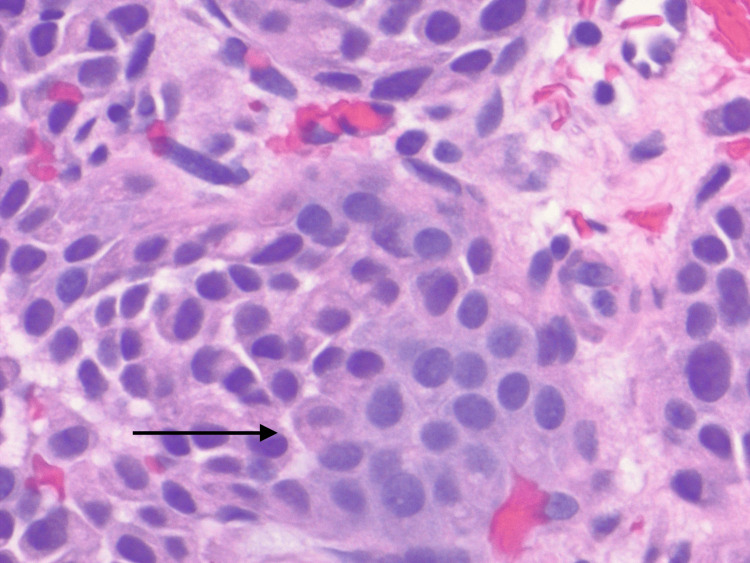
Biopsy demonstrating neuroendocrine tumor cells (400x magnification)

Targeted cold forceps biopsies were obtained from the areas of abnormal mucosa. Histopathologic evaluation demonstrated a well-defined submucosal proliferation of small, monomorphic cells with round nuclei, granular chromatin, and scant cytoplasm arranged in trabecular and glandular patterns. No necrosis, pleomorphism, or vascular invasion was identified. The mitotic rate was less than two per 10 high-power fields. Adjacent mucosa showed preserved villous architecture with acute and chronic inflammatory changes, including surface erosion and focal crypt abscess formation. Immunohistochemical staining showed diffuse positivity for synaptophysin and negativity for chromogranin A. The Ki-67 proliferation index was less than 1%, consistent with a well-differentiated, grade 1 neuroendocrine tumor of the duodenum (Figures 2, 3). 

**Figure 4:**
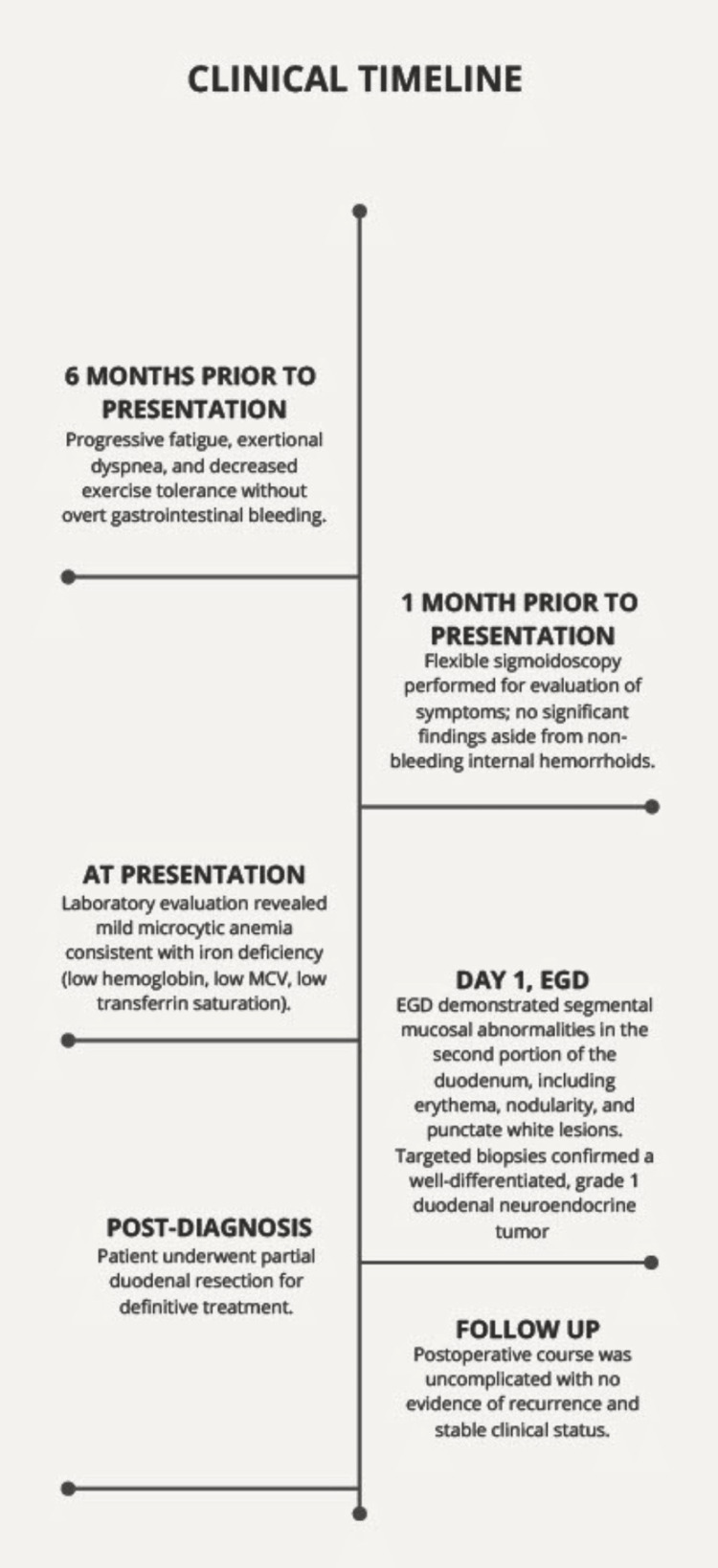
Clinical timeline Clinical timeline illustrating symptom progression, diagnostic evaluation, treatment, and follow-up. EGD = esophagogastroduodenoscopy; MCV = mean corpuscular volume

Given these findings, the patient underwent partial duodenal resection at an outside facility. His postoperative course was uncomplicated, and he was maintained on proton pump inhibitor therapy. On follow-up, he remained clinically stable with no evidence of recurrence. The patient’s clinical course, including symptom onset, diagnostic evaluation, and management, is summarized in Figure 4. 

## Discussion

Duodenal neuroendocrine tumors are rare gastrointestinal neoplasms, representing a small fraction of all neuroendocrine tumors, and are most often identified incidentally during endoscopic evaluation [1,2]. Although typically indolent, they may present with nonspecific symptoms, and less commonly, with iron deficiency anemia secondary to chronic, often occult gastrointestinal blood loss [3-5]. This makes diagnosis particularly challenging, as more common etiologies of anemia are usually prioritized in the initial evaluation.

In this case, the patient presented with symptomatic iron deficiency anemia without overt gastrointestinal bleeding, prompting endoscopic evaluation. The absence of a clear bleeding source highlights the importance of maintaining a broad differential diagnosis when evaluating anemia in older adults [6]. While duodenal mucosal abnormalities such as erythema, friability, and nodularity are frequently attributed to inflammatory processes, these findings may also reflect underlying neoplastic pathology. As demonstrated here, careful mucosal inspection and targeted biopsy were essential in establishing the diagnosis.

Histologically, duodenal neuroendocrine tumors are characterized by uniform cells with round nuclei, finely granular chromatin, and a trabecular or nested growth pattern [7,8]. Immunohistochemical staining plays a critical role in confirming neuroendocrine differentiation, with synaptophysin serving as a sensitive marker [9]. Chromogranin expression may be variable and is not required for diagnosis, particularly in well-differentiated, low-grade tumors [7]. Tumor grading, based on mitotic activity and Ki-67 proliferation index, remains central to prognostication, with grade 1 lesions demonstrating low proliferative activity and favorable outcomes [7,8].

The incidence of neuroendocrine tumors has increased over recent decades, likely due to improved detection and surveillance practices [2,7]. As a result, clinicians are more frequently encountering these tumors in a variety of clinical contexts. Despite this trend, duodenal neuroendocrine tumors remain uncommon, and their presentation with iron deficiency anemia is rare [3-5]. Therefore, the importance of recognizing that even indolent and rare tumors may contribute to chronic blood loss should be considered when routine evaluations do not identify a clear etiology.

Management of duodenal neuroendocrine tumors depends on tumor size, depth of invasion, and histologic grade. Small, well-differentiated lesions are often amenable to endoscopic resection, while larger or more invasive tumors may require surgical intervention [10,11]. In this case, the patient underwent partial duodenal resection with an uncomplicated postoperative course and no evidence of recurrence on follow-up, consistent with the favorable prognosis associated with low-grade disease [11].

Several limitations should be acknowledged in this case. A complete anemia evaluation, including additional hematologic studies such as ferritin, vitamin B12, folate, and reticulocyte count, was not fully available for review. In addition, cross-sectional imaging results were unavailable, and a complete lower gastrointestinal evaluation with colonoscopy had not been performed at the time of diagnosis, as only prior flexible sigmoidoscopy findings were available. These factors limit the ability to definitively attribute the patient’s mild iron deficiency anemia solely to the duodenal neuroendocrine tumor. Another limitation is the absence of intraoperative imaging and decision-making prior to the surgery, as the surgical procedure was performed at an outside institution.

Overall, this case highlights an uncommon presentation of a rare tumor and reinforces the importance of comprehensive endoscopic evaluation in patients with unexplained iron deficiency anemia. Early recognition and histologic confirmation are essential, as timely intervention is associated with excellent outcomes.

## Conclusions

Duodenal neuroendocrine tumors may present with subtle mucosal abnormalities and occult iron deficiency anemia without an identifiable bleeding source. This case emphasizes the importance of maintaining a broad differential diagnosis and performing careful endoscopic inspection with targeted biopsy when evaluating unexplained anemia. Recognition of these atypical presentations allows for timely diagnosis and effective management of rare gastrointestinal malignancies.
